# Semi-Automated Microfluidic Device Combined with a MiniPCR-Duplex Lateral Flow Dipstick for Screening and Visual Species Identification of Lymphatic Filariae

**DOI:** 10.3390/mi13020336

**Published:** 2022-02-21

**Authors:** Achinya Phuakrod, Navapon Kusuwan, Witsaroot Sripumkhai, Pattaraluck Pattamang, Sirichit Wongkamchai

**Affiliations:** 1 Department of Parasitology, Faculty of Medicine Siriraj Hospital, Mahidol University, Bangkok 10700, Thailand; achinyalek@hotmail.com; 2 Graduate Program in Medical Technology, Faculty of Alliance Health Sciences, Thammasat University, Pathum Thani 12120, Thailand; navaponkusuwan@hotmail.com; 3 Thai Microelectronic Center, National Electronics and Computer Technology Center, Thailand Science Park, Pathum Thani 12120, Thailand; witsaroot.sripumkhai@nectec.or.th (W.S.); pattaraluck.pattamang@nectec.or.th (P.P.)

**Keywords:** semi-automated microfluidic device, miniPCR-duplex lateral flow dipstick, mass screening, visual species identification, lymphatic filariae, *B. malayi*, *W. bancrofti*

## Abstract

Lymphatic filariasis (LF) is a leading cause of permanent disability worldwide that has been listed as a neglected tropical disease by the World Health Organization. Significant progress made by the Global Program to Eliminate Lymphatic Filariasis (GPELF) has led to a substantial decline in the population of the worm that causes LF infection. Diagnostic assays capable of detecting low levels of parasite presence are needed to diagnose LF. There is also a need for new tools that can be used in areas where multiple filarial species are coendemic and for mass screening or for use in a point-of-care setting. In the present study, we applied our previously developed semi-automated microfluidic device in combination with our recently developed mini polymerase chain reaction (miniPCR) with a duplex lateral flow dipstick (DLFD) (miniPCR-DLFD) for rapid mass screening and visual species identification of lymphatic filariae in human blood. The study samples comprised 20 *Brugia malayi* microfilariae (mf) positive human blood samples, 14 *Wuchereria bancrofti* mf positive human blood samples and 100 mf negative human blood samples. Microfilariae detection and visual species identification was performed using the microfluidic device. To identify the species of the mf trapped in the microfluidic chips, DNA of the trapped mf was extracted for miniPCR amplification of *W. bancrofti* and *B. malayi* DNA followed by DLFD. Thick blood smear staining for microfilariae detection was used as the gold standard technique. Microfilariae screening and visual species identification using our microfluidic device plus miniPCR-DLFD platform yielded results concordant with those of the gold standard thick blood smear technique. The microfluidic device, the miniPCR and the DLFD are all portable and do not require additional equipment. Use of this screening and visual species identification platform will facilitate reliable, cost-effective, and rapid surveillance for the presence of LF infection in resource-poor settings.

## 1. Introduction

Lymphatic filariasis (LF) is a mosquito-borne, globally distributed tropical disease that can cause permanent disability and is a significant threat to public health worldwide. A variety of assays are available for diagnosing and/or monitoring LF infection, including thick blood smear staining for microfilaria detection, antigen and antibody detection testing and parasite nucleic acid detection testing [1]. The microfilaria detection using the thick blood smear staining technique, although it is easy and cheap to perform, cannot be accomplished within one day because the drying step of the smear slide requires 12–48 h, according to the World Health Organization’s procedure [2], thus, it is not suitable to be a point of care testing. Moreover, a high number of slides to be examined under the microscope may lead to incorrect diagnoses when fatigue occurs [3].

Progress made by the Global Program to Eliminate Lymphatic Filariasis (GPELF) has led to a substantial decrease in the levels of infection and the rates of disease transmission [4]. However, as the rates of disease-related measurement parameters continue to decrease, more sensitive diagnostic assays that are capable of detecting low levels of parasite presence are needed. Recent studies reported the need for new tools with greater specificity and sensitivity, especially in areas where multiple filarial species are co-endemic, for mass screening or for use in a point-of-care setting [5,6].

The processing capacity of laboratories is limited because highly trained personnel must supervise the analysis and the biological sample must be transported from the patient at the point of collection to the lab and the results are usually obtained ≥24 h later [7]. Furthermore, laboratory-based diagnosis cannot meet the demands of mass identification of pathogens [8,9,10].

Many methods have been proposed to deliver low-cost and effective diagnosis of infectious diseases. Microfluidic platforms require and consume only minute amounts of samples and reagents, which minimizes waste and expense, and they offer the unique physical advantage of microscale fluid flow [11]. Innovations in microfluidic technologies have facilitated the development of portable, robust, accurate, and sensitive diagnostic devices for point-of-care use [10]. A microfluidic chip, which is often referred to as a lab on a chip, is a chip that was developed for use in life science and biology that is characterized by fluid flow along micro-scale channels (i.e., microfluidics) [12].

Lateral flow technology is robust and inexpensive and does not require an additional source of electricity, cold storage/transport, or specialized reagents [8]. The immunochromatographic assay (ICT), which is also known as a lateral flow immunoassay (LFIA) or strip test, is a paper-based micromachine that is used for detection and quantification of analytes in complex mixtures. The sample is deposited onto the test device and the results are displayed within 5–30 min. The ICT has rapid, simple, cost-effective, specific, and sensitive characteristics [13,14,15], is user-friendly and is widely applied for the detection of bacterial, viral, and parasitic diseases [16,17].

In this study, we integrated our previously developed semi-automated microfluidic device [12] with our recently developed mini polymerase chain reaction (miniPCR) and duplex lateral flow dipstick (DLFD) [18] to create a miniPCR-DLFD platform for mass screening and visual species identification of lymphatic filariae (*W. bancrofti* and *B. malayi*) in human blood samples.

## 2. Materials and Methods

### 2.1. Ethics Approval

The protocol for this study was approved by the Siriraj Institutional Review Board (SIRB) of the Faculty of Medicine Siriraj Hospital, Mahidol University, Bangkok, Thailand (approval no. Si129/2016). This study complied with all ethical principles and guidelines for human experimentation set forth by the National Research Council of Thailand and written informed consent to participate was obtained from all enrolled study subjects.

### 2.2. Study Samples

The study samples comprised 20 *Brugia malayi* microfilariae (mf) positive human blood samples, 14 *Wuchereria bancrofti* mf positive human blood samples, and 100 mf negative blood samples obtained from healthy human subjects.

### 2.3. Thick Blood Smear Staining for Microfilariae Detection

Thick blood smear staining for mf detection was performed using ethylenediaminetetraacetic acid (EDTA) blood obtained from study samples according to the standard World Health Organization (WHO) procedure [19]. Briefly, 50 µL of EDTA blood was deposited onto a clean glass slide and then the blood was spread to create a circular smear. The blood smear was then allowed to dry overnight at room temperature (RT). The dried blood smear was immersed in freshly prepared working Giemsa stain for 45–60 min. The slide was then removed and rinsed by dipping 3 to 4 times in Giemsa buffer. After air-drying, the slides were examined under a microscope (100×) for detection and species identification of mf.

### 2.4. Microfluidic Device for Detection of Microfilariae from Human Blood Sample

**Figure 1:**
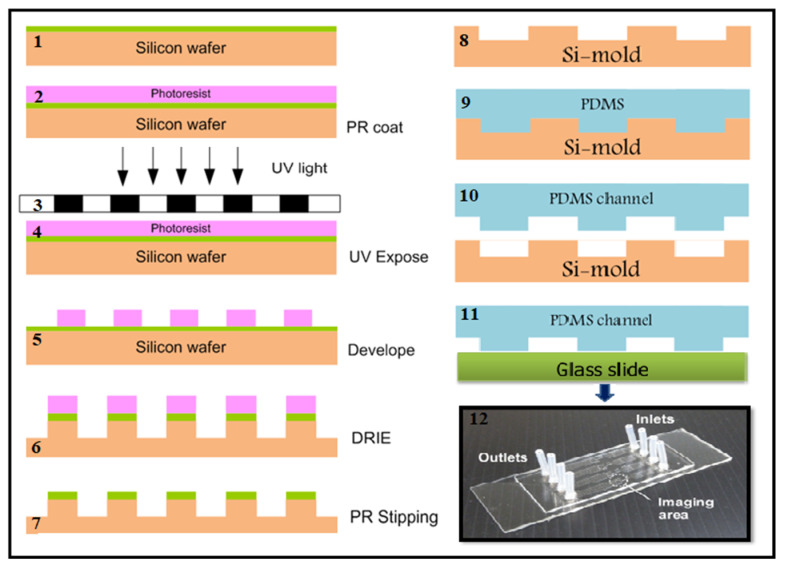
Diagram of the silicon-mold creating (**1**–**7**); polydimethylsiloxane (PDMS) casting (**8**–**10**); PDMS channel and glass slide bonding (**11**) and the fabricated microfluidic chip (**12**).

Detection for microfilariae was performed in all study samples using the semi-automated microfluidic device, as described previously [12]. The microfluidic chip was developed according to the diagrams shown in Figure 1. Briefly, 50 μL of blood was suspended in 150 μL of lysis buffer A, mixed, and then incubated at RT for 10 min. The prepared sample was drawn into a syringe followed by the drawing of 15 μL of buffer B into the same 1 mL syringe that connects to the adapter and the inlet port. The pump was started, and the sample solution was introduced into the microfluidic device via the inlet port. If any microfilariae were present, they were trapped in the microfluidic chip while the remaining solution exited the microfluidic chip via the outlet port and into the waste tube. Species identification of the mf trapped in the microfluidic chip was performed using our previously developed miniPCR-DLFD [18].

### 2.5. Extraction of DNA from the Trapped Microfilariae

To identify the species of the mf trapped in the microfluidic chips, 100 μL Tris-EDTA buffer was dispensed into the chip via the inlet port. The chip was placed on a hot plate at 56 °C for 15 min after which the solution containing the trapped mf was withdrawn through the outlet port and transferred to a 1.5 mL Eppendorf tube. The tube was subjected to centrifugation at 15,000× *g* for 10 min. The supernatant was discarded, and the DNA was extracted from the pelleted material using a Roche High Pure PCR Template Preparation Kit, according to the manufacturer’s instructions (Roche Diagnostics GmbH, Penzberg, Germany). Following extraction, the DNA was eluted in 100 µL of elution buffer and stored at −20 °C until use. The DNA concentration was determined using a Nanodrop 1000 spectrophotometer (Thermo Fisher Scientific, Inc., Waltham, MA, USA) before the DNA was subjected to PCR and miniPCR analysis.

### 2.6. MiniPCR for Amplification of W. bancrofti and B. malayi DNA

Species identification was performed via HhaI/SspI gene amplification using our recently developed miniPCR (Amplyus) [18]. The primer designs were based on the alignments of the HhaI and SspI repetitive non-coding DNA sequences of *B. malayi* and *W. bancrofti*, respectively. The presence of the 145-bp and 182-bp amplification products of HhaI and SspI indicated the presence of *B. malayi* and *W. bancrofti*, respectively. Since we used the DLFD to detect the PCR amplified product, the 5′ ends of the designed forward primers for HhaI and SspI were labeled with fluorescein isothiocyanate (FITC), whereas the 5′ ends of the reverse primers for HhaI and SspI were labeled with digoxin (DIG) and biotin, accordingly [18]. The PCR amplifications were performed using a miniPCR instrument (DBA miniPCR bio; Amplyus LLC., Cambridge, MA, USA). In 20 µL of a reaction mix composed of PCR Master Mix (Quantabio, Beverly, MA, USA) 10 µL, 0.2 µM of each forward and reverse primer, distilled water (dH2O) 7.2 µL, and, DNA template 2 µL. DNA from blood samples containing mf of *B. malayi* and mf of *W. bancrofti* were used as positive controls and nuclease-free water as a negative control. PCR assays for *B. malayi* and *W. bancrofti* were separately performed employing the reagents and conditions, as mentioned above.

### 2.7. Lateral Flow Dipstick (DLFD) for Detection of Amplification Products

**Figure 2:**
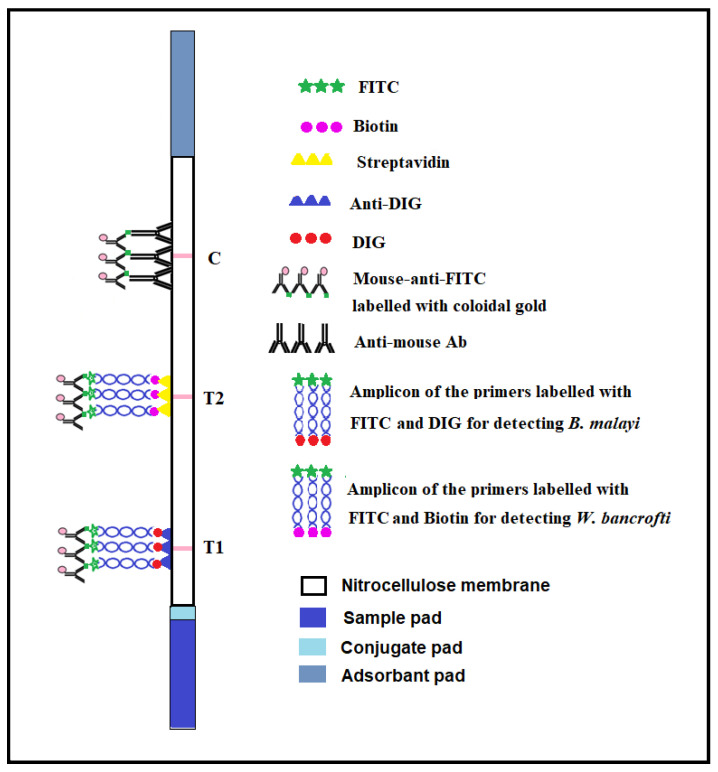
Schematic diagram reveals the principle of the duplex lateral flow dipstick (DLFD) for lymphatic filariae DNA detection.

A duplex lateral flow dipstick (DLFD) that was recently developed by our group was used for species identification of the amplicon [18]. Briefly, 1 µL of amplification product from the miniPCR amplification was added into a well of a 96-well plate containing 100 µL of sample buffer. The dipstick was placed into the well vertically and left for 10 min. The appearance of a pink-colored line on both the test and control lines that was observable to the naked eye indicated a positive test result. A pink-colored line appearing only on the control line indicated a negative test result. A diagram demonstrating the function of the DLFD is shown in Figure 2.

## 3. Results

**Figure 3:**
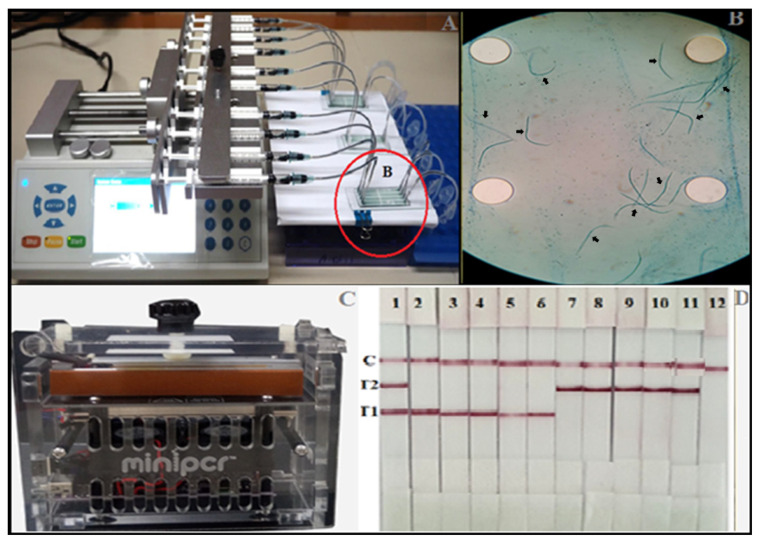
Microfluidic device coupled with the miniPCR-DLFD for rapid detection and identification of microfilariae of lymphatic filariae in human blood samples. (**A**) Microfluidic device. (**B**) Microfilariae trapped in the microfluidic chip (arrows). (**C**) The miniPCR thermocycler and (**D**) reaction of DLFD strips for species identification of the amplicon from trapped microfilariae (1 µL of each amplicon/strip). Strip 1: Positive control containing *W. bancrofti* and *B. malayi* DNA, strips 2–11: Representative of test samples, strip 12: Negative control.

The 100 mf negative blood samples were confirmed negative by no mf detection by either thick blood smear staining or by the microfluidic device. For all 34 mf positive blood samples tested, mf from all samples was trapped in the detection zone of the microfluidic chip. Species identification of the trapped mf was performed by miniPCR thermocycler followed by DLFD. Of the 34 mf positive samples, 14 mf were identified as *W. bancrofti*, and 20 mf were identified as *B. malayi*. All mf positive blood samples showed concordant results for both mf detection and species identification between gold standard blood smear staining and our testing platform consisting of the microfluidic device and the miniPCR-DLFD (Table 1). Figure 3 shows the microfluidic device, the miniPCR thermocycler and a representative reaction of the amplicon of the trapped microfilariae using the DLFD.

## 4. Discussion

Lymphatic filariasis has historically been endemic in Thailand. In 2001, the Thailand Ministry of Public Health set up the Migrant Health Insurance Scheme for all documented and undocumented migrants who are not covered by social health insurance to cover mandatory health screening at first entry and subsequent yearly renewals of the residence permit [6], including screening for *W. bancrofti* mf (mf challenge test with diethylcarbamazine [DEC]. Every year, approximately one million registered migrant workers are screened for microfilariae using thick blood smear staining [6]. However, screening for mf using the thick blood smear staining technique is not practical for a very large number of blood samples because it is beyond the processing capacity of most laboratories. Moreover, manual visual evaluation of a large number of stained slides under a microscope causes eye fatigue and dizziness in many operators, and this may adversely influence the accuracy and efficiency of testing [12].

We previously developed a semi-automated microfluidic device that is more efficient than the thick blood smear staining technique in some aspects. By way of example, our previously developed microfluidic device yields more rapid, higher-throughput detection of microfilariae. It requires approximately 5.7 min to complete one blood sample, 15 min to prepare 10 samples, 37 min to perform the testing, and 5 min for mf observation under the microscope [12]. Moreover, the mean ± SD and % coefficient variation (%CV) of high, moderate, and low levels of mf/ 50 µL in intra-assay were 27.22 + 2.94, 10.8%; 15.44 ± 1.3, 8.42%, and 5.25 ± 0.5, 9.38%, respectively. While the mean ± SD and %CV of high, moderate, and low levels of mf/50 µL in inter-assay were 31 ± 1.22, 4.44%; 15.6 ± 0.89, 4.16%, and 5.8 ± 0.45, 4.66%, accordingly. It revealed high efficacy and reproducibility of the microfluidic device [12]. We also applied our developed microfluidic device for investigation on the prevalence of canine microfilaremia [19]. However, our microfluidic device requires an expensive specialized instrument (i.e., Light Cycler LC480 to perform a high-resolution melt (HRM) real-time PCR) for species identification of the trapped mf [12,19].

Standard conventional PCR is one of the most widely used techniques for the diagnosis of many infectious diseases, including filariasis [4,20]. However, the use of standard PCR is limited by its requirement for dedicated laboratory instruments, and it is only available at some facilities. The miniPCR thermocycler is an attractive and comparatively affordable device with the potential for use at point-of-collection sites since it has a user-friendly format, it is cheap and portable [21,22]. However, the amplicon from the miniPCR still requires gel electrophoresis and imaging for species identification. The use of a PCR-nucleic acid lateral flow immunochromatographic assay (PCR-NALFIA) using a lateral flow dipstick (LFD) assay which can detect specific DNA products in as little as 10–15 min can conquer the limitation [23,24]. The miniPCR-DLFD platform exhibited a detection limit of 2 and 4 mf per milliliter (mL) of blood for *B. malayi* as well as *W. bancrofti*, respectively, and no cross-amplification was observed with other parasites, demonstrating its high sensitivity and specificity [18].

Recent technical advances permit multiplexing by spatial separation of lines on a single strip. The multiplex detection of more than one species of the pathogen on a single strip offers the advantages of increased speed and lower cost. Multiplex rapid lateral flow diagnostics offers the potential of distinguishing among multiple pathogens, thereby facilitating diagnosis and improving patient care [8,10,18].

In this study, we applied our previously developed semi-automated microfluidic device to screen for mf of lymphatic filariae in human blood. We also replaced the use of HRM real-time PCR with our recently developed miniPCR-DLFD for rapid and visual species identification of the trapped mf. Although we included a relatively small number of infected blood samples, the results from our studied combination test platform were concordant with those obtained from the gold standard thick blood smear technique. The benefit of detecting parasite DNA using the miniPCR thermocycler followed by the DLFD is rapid and simple visual detection and species identification of the trapped mf in as little as 15 min.

## 5. Conclusions

We applied our previously developed semi-automated microfluidic device in combination with our recently developed miniPCR-DLFD for LF detection and visual species identification. Microfilariae screening and visual species identification using our microfluidic device plus miniPCR-DLFD platform yielded results concordant with those of the gold standard thick blood smear technique. Using this novel combination testing platform, a larger number of blood samples can be processed in point-of-collection settings at a modest cost. This new diagnostic tool, which is portable and not dependent on cumbersome support equipment or an external electricity supply, may contribute to lower cost, faster and less labor-intensive diagnosis of LF and other currently neglected tropical diseases.

## Figures and Tables

**Table 1:** Microfilaria detection and species identification in 134 human blood samples using the microfluidic device-iminiPCR-DLFD platform and microscopy.

Mf Detection and Species Identification by Microfluidic Device and miniPCR-DLFD	Mf Detection and Species Identification by Microscopy (Giemsa Staining)			Number of Samples
	*B. malayi*	*W. bancrofti*	Negative	
*B. malayi*	20	0	0	20
*W. bancrofti*	0	14	0	14
inegative	0	0	100	100
Total	20	14	100	134
